# Histopathological investigation in porcine infected with torque teno sus virus type 2 by inoculation

**DOI:** 10.1186/1743-422X-8-545

**Published:** 2011-12-15

**Authors:** Miao Mei, Ling Zhu, Yun Wang, Zhiwen Xu, Ling Zhao, Xi Peng, Yunfei Wu, Song Li, Wanzhu Guo

**Affiliations:** 1 Animal Biotechnology Center, College of Veterinary Medicine of Sichuan Agricultural University, Ya' an 625014, P R China; 2 Key Laboratory of Animal Disease and Human Health, College of Veterinary Medicine of Sichuan Agricultural University, Ya' an 625014, P R China

**Keywords:** Torque teno sus virus 2(TTSuV2), Porcine, Histopathological lesions, Hematoxylin and eosin

## Abstract

**Background:**

Porcine torque teno sus virus (TTSuV) is a small icosahedral and non-enveloped virus which contains a single-stranded (ssDNA), circular and negative DNA genome and infects mainly vertebrates and is currently classified into the 'floating' genus Anellovirus of Circoviridae with two species. Viral DNA of both porcine TTSuV species has a high prevalence in both healthy and diseased pigs worldwide and multiple infections of TTSuV with distinct genotypes or subtypes of the same species has been documented in the United States, Europe and Asia. However, there exists no information about histopathological lesions caused by infection with porcine TTSuV2.

**Methods:**

Porcine liver tissue homogenate with 1 ml of 6.91 × 10^7^genomic copies viral loads of porcine TTSuV2 that had positive result for torque teno sus virus type 2 and negative result for torque teno sus virus type 1 and porcine pseudorabies virus type 2 were used to inoculate specific pathogen-free piglets by intramuscular route and humanely killed at 3,7,10,14,17,21 and 24 days post inoculation (dpi), the control pigs were injected intramuscularly with 1 ml of sterile DMEM and humanely killed the end of the study for histopathological examination routinely processed, respectively.

**Results:**

All porcine TTSuV2 inoculated piglets were clinic asymptomatic but developed myocardial fibroklasts and endocardium, interstitial pneumonia, membranous glomerular nephropathy, and modest inflammatory cells infiltration in portal areas in the liver, foci of hemorrhage in some pancreas islet, a tiny amount red blood cells in venule of muscularis mucosae and outer longitudinal muscle, rarely red blood cells in the microvasculation and infiltration of inflammatory cells (lymphocytes and eosinophils) of tonsil and hilar lymph nodes, infiltration of inflammatory lymphocytes and necrosis or degeneration and focal gliosis of lymphocytes in the paracortical zone after inoculation with porcine TTSuV2-containing tissue homogenate.

**Conclusions:**

Analysis of these presentations revealed that porcine TTSuV2 was readily transmitted to TTSuV-negative swine and that infection was associated with characteristic pathologic changes in specific pathogen-free piglets inoculated with porcine TTSuV2. Those results indicated no markedly histopathological changes happened in those parenchymatous organs, especially the digestive system and immune system when the specific pathogen-free pigs were infected with porcine TTSuV2, hence, to some extent, it was not remarkable pathological agent for domestic pigs at least. So, porcine TTSuV2 could be an unrecognized pathogenic viral infectious etiology of swine. This study indicated a directly related description of lesions responsible for TTSuV2 infection in swine.

## Background

In 1997, torque teno sus virus (TTSuV) was discovered in Japan in a patient with post-transfusion hepatitis of unknown aetiology [1]. Torque teno sus virus (TTSuV) is a small icosahedral and non-enveloped virus which contains a single-stranded (ssDNA), circular and negative DNA genome and infects mainly vertebrates, such as human, non-primate and domestic species, including domestic swine and wild boar [2-8]. In 2005, torque teno sus virus was firstly classified into the 'floating' genus Anellovirus of Circoviridae by the International Committee on Taxonomy of Viruses, suggesting the new and present name for TTSuV [4,9], and was firstly described as the homologous counterpart of the human TTSuV from domestic pigs in Japan in 2002 [8].

Recently, TTSuVs have attracted markedly interest within the research community [4] and porcine TTSuVs have been detected using PCR assays in pig populations from the United States, Canada, Brazil, Spain, France, Italy, Germany, China, Thailand, Korea, Hungary, Australia and Cuban [10-21], with variable prevalence, those results have determined that porcine TTSuVs are ubiquitous and widely distributed in the world. A recent retrospective study revealed evidence of both genogroups of porcine TTSuV infection in pigs as early as 1985 in Spanish intensive conventional commercial pig farms [22]. In spite of being a single strand DNA virus, the sequences of human TTSuV genome are markedly diverse, containing five groups and 34 genotypes [4,23,24]. The genome of porcine TTSuV is approximately 2.8 kb in length and contains three or four overlapping open reading frames (ORFs) as well as a short stretch of untranslated region with high GC content [25] and investigations in swine have identified two distinct TTSuV genogroups for TTSuV-1 and TTSuV-2 [12]. At present, both genogroups have been defined as species [26].

To date, much attention has been paid to TTSuV infection in other vertebrates [8,27,28], especially pigs [4,10,13,15-17,19-22,29-33]. Even though porcine TTSuVs are ubiquitous in swine, the pathogenesis is not clear [4]. Despite the fact human TTSuV infection is not considered to be directly associated with a specific disease [34], porcine TTSuV has been proven to partially contribute to the experimental induction of porcine dermatitis and nephropathy syndrome (PDNS) associated with the porcine reproductive and respiratory syndrome virus (PRRSV) infection [35], and postweaning multisystemic wasting syndrome (PMWS) associated with PCV-2 infection in a gnotobiotic pig model [36]. Moreover, high prevalence of TTSuVs, especially TTSuV2, have been detected in PMWS pigs [18]. These results suggest that porcine TTSuVs are probably pathogenic in pigs due to synergistic effects with together different viruses acting. However, further studies will be required to associate TTSuVs infection with specific diseases.

No tissue culture system for TTSuVs propagation has been identified [4]. Although porcine TTSuV is not found to be associated with any swine disease, coinfection of pigs with TTSuV and other known swine pathogens (PMWS, PCV2, PRRSV, PRV) may result in enhanced disease [16,18,31,36-42]. There are also concerns for risk of potential human infection during xenotransplantation and a public health problem. The role of the sows in transmitting porcine TTSuV to piglets and the infection dynamics of both swine TTSuV genogroups (TTSuV1 and TTSuV2) during the lactation period has been studied [29,33]. However, the resent studies is roughly focused on serum sample analysis of sows and piglets based on porcine torque teno virus nucleic acid by conventional nested PCR and real-time PCR assays [18,19,26] and no methods about tissue detection, pathogenesis or cell culture lines studies were conducted, and nothing is known regarding porcine TTSuV2-specific histopathological examination, except that the development of ELISA assay based on expressing of the putative OFR1 capsid protein of PTTSuV2 for the possibility of serological diagnoses [43,44], The aim of this study was to describe whether the presence of specific histopathological lesions of PTTSuV-2 infection was observed by hematoxylin-eosin stain and olympus light microscope on an intensive pig farm in Sichuan of China.

## Results

**Figure 1:**
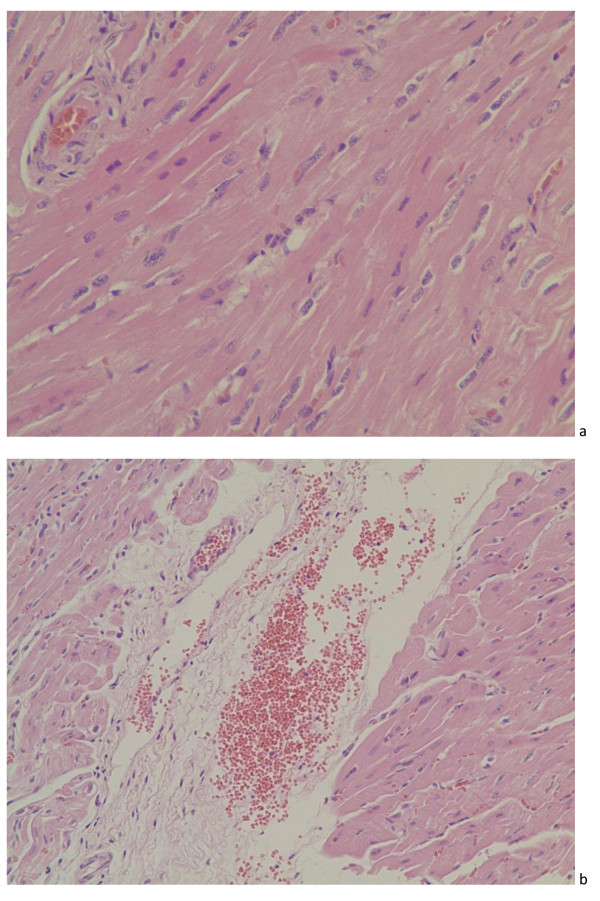
**Photomicrograph of heart from two of the pigs**. There is hyperemia in artery and subtle congestion among myocardial fibroklasts at 17 dpi. (HE. ×400), and large number of red blood cells distribute in endocardium, but cells of endocardium were not found changes and lesions from a specific pathogen-free pig euthanized at 14 dpi. with the liver homogenate of porcine TTSuV2 (HE. ×200). Those histopathological lesions were marked with arrowheads.

Control animals displayed no clinical manifestations throughout the study. No obvious gross lesions were observed between the control group and the infected animals on necropsy examination. In totally, specific microscopical lesions were found in the tissues from the twenty-one pigs infection with porcine torque teno sus virus 2 throughout the study, and there existed subtle pathologic changes in the tissues containing myocardial fibroklasts and endocardium, interstitial pneumonia, membranous glomerular nephropathy, and modest inflammatory cells infiltration in portal areas in the liver, foci of hemorrhage in some pancreas islet, a tiny amount red blood cells in venule of muscularis mucosae and outer longitudinal muscle, rarely red blood cells in the microvasculation and infiltration of inflammatory cells (lymphocytes and eosinophils) of tonsil and hilar lymph nodes, infiltration of inflammatory lymphocytes and necrosis or degeneration and focal gliosis of lymphocytes in the paracortical zone after inoculation with the porcine TTSuV2-containing tissue homogenate; these changes were not detected in uninoculated control pigs or pigs injected with tissue homogenate devoid of porcine TTSuV2 genome. Firstly, hyperemia and congestion were found in the myocardial fibroklasts and endocardium seen in Figure 1a and 1b.

**Figure 2:**
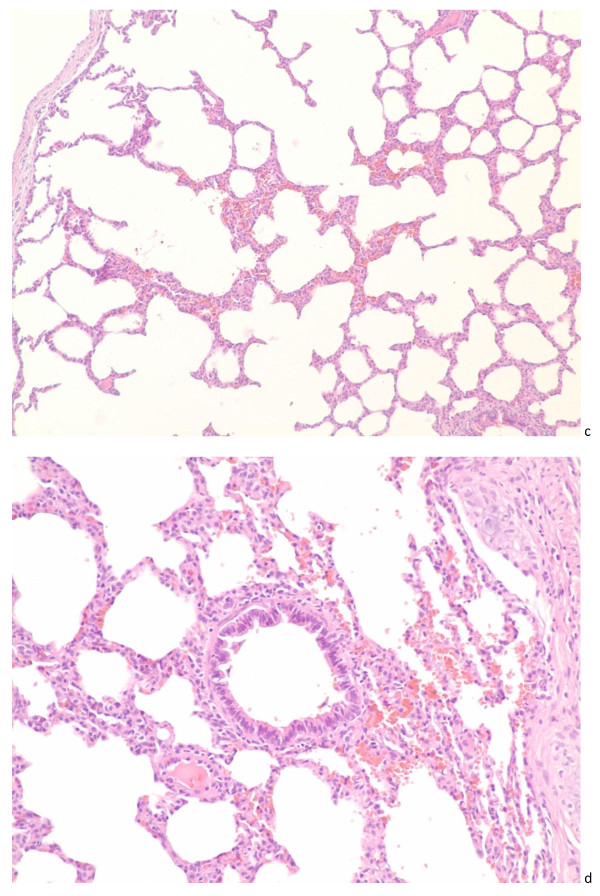
**The lung lesions contained of a mild interstitial pneumonia**. The lung lesions contained of a mild interstitial pneumonia characterized by a slight thickening of the alveolar septa by mononuclear cells and congestion, and bronchial epithelial cells happened defluvium and inflammation infiltration in pulmonary alveolus(c, 10 dpi. HE. ×100; d,24 dpi HE. ×200).

The lung lesions contained of a mild interstitial pneumonia characterized by a slight thickening of the alveolar septa by mononuclear cells and congestion, and bronchial epithelial cells happened defluvium and inflammation infiltration in pulmonary alveolus seen in Figure 2c and 2d.

**Figure 3:**
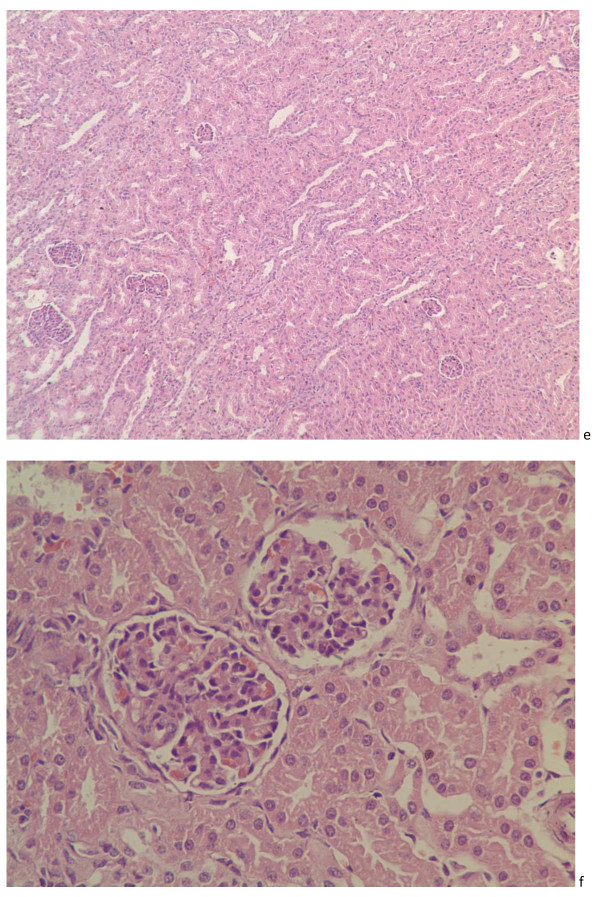
**Sections of porcine kidney**. Sections of porcine kidney showed slight congestion in the renal tube at 7 dpi. (e, HE. ×100), the degeneration and necrosis in renal capsular epithelial cells and the ventral cells defluvium of renal capsule in capsular space at 17 dpi (f. HE. ×400).

Figure 3e and 3f illustrated the histopathological findings in the kidney infected with PTTSuV2, slight congestion happened in the retal tube and the degeneration and necrosis or degeneration and focal gliosis were observed in epithelial cells, that's to say membranous glomerular nephropathy. In addition, the ventral cells defluvium of renal capsule were observed in capsular space.

**Figure 4:**
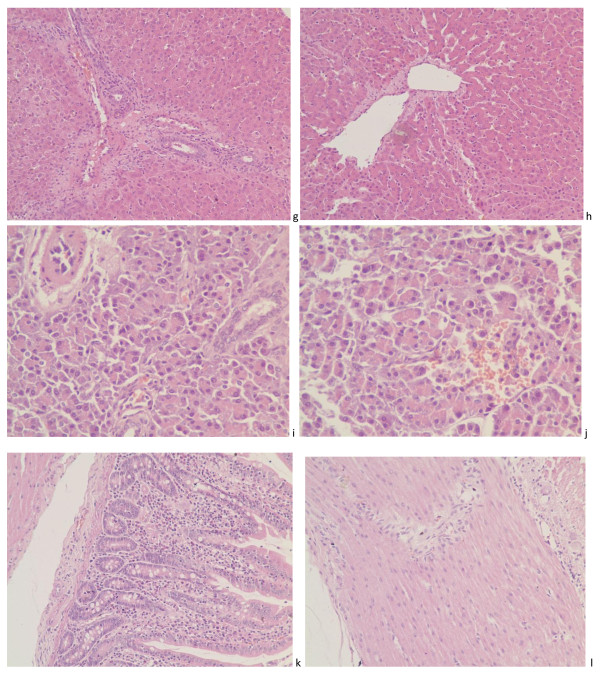
**Histopathological observation of the digestive system**. Histopathological observation of the digestive system including live, pancreas and duodenum: (**g**) showed that there existed a tiny amount of red blood cells in the interlobular veins and sinusoid and a bit of inflammatory cells infiltration in portal areas from a specific pathogen- free piglets killed at 10 dpi. (HE. ×200), but no various pathological lesions were observed at other parts of the liver at 10 dpi. (h. HE. ×200); (**i**) and (**j**) manifested subtle congestion among pancreas islets and foci of hemorrhage in some pancreas islet, a little of infiltration was inferred as pancreatic juice or electrolyte inner interlobular duct at 21 dpi. (HE. ×200); however, non-specific histopathological lesions of the duodenum except a tiny amount red blood cells in venule of muscularis mucosae and outer longitudinal muscle was observed and could be pictured as normal histological graph (k, at 3 dpi. HE. ×200; l, at 24 dpi, HE. x200).

Figure 4g, h, i, j, k and 4l represented the histopathological changes in the digestive system containing liver, pancreas and duodenum. The sections of liver showed that there existed a tiny amount of red blood cells in the interlobular veins and sinusoid and a bit of inflammatory cells infiltration in portal areas, but no various pathological lesions were observed at other parts of the liver; the photomicrograph of pancreas manifested subtle congestion among pancreas islets and foci of hemorrhage in some pancreas islet, a little of infiltration was inferred as pancreatic juice or electrolyte inner interlobular duct; however, non-specific histopathological lesions of the duodenum except a tiny amount red blood cells in venule of muscularis mucosae and outer longitudinal muscle was observed and could be pictured as normal histological graph.

**Figure 5:**
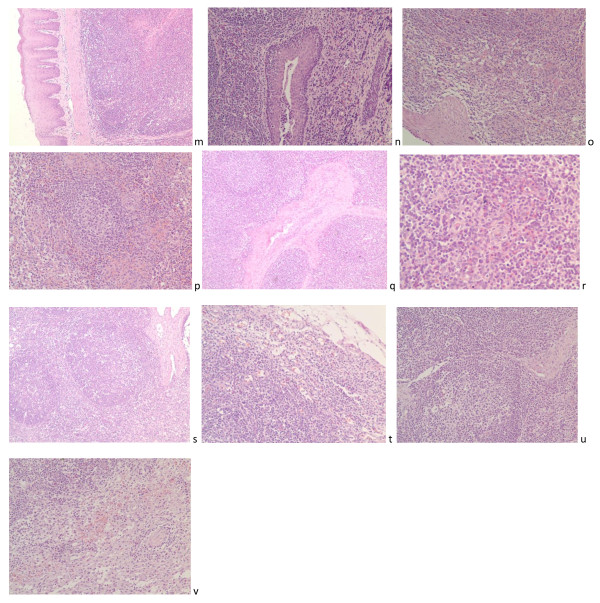
**Photomicrograph of the main immune system**. Photomicrograph of the main immune system involving in spleen, tonsil, hilar lymph nodes, mesenteric lymph nodes and inguinal lymph nodes collected from experimental groups weaned piglets euthanized at different days post inoculation porcine TTSuV2. With the development of infection, the sections of tonsil, spleen, hilar lymph nodes and mesenteric lymph nodes showed the normal architecture with no special lesions through the study but only rarely red blood cells in the microvasculation and infiltration of inflammatory cells (lymphocytes and eosinophils) of tonsiland hilar lymph nodes marked by black arrowheads (m, o, p, s. HE. ×100; n, t. HE. ×200; r. HE. ×400). In addition, inguinal lymph nodes' architecture was normal, but congestion, infiltration of inflammatory lymphocytes and necrosis or degeneration and focal gliosis of lymphocytes in the paracortical zone were observed at 24 dpi. (u. HE. ×100; v. HE. ×200). The red four-point star, five-point star and rhomboid represent the normal architecture of those immune organs.

The micrographic pathological changes of the immune system showed in Figure 5m, n, o, p, q, r, s, t, u and 5v involving in spleen, tonsil, hilar lymph nodes, mesenteric lymph nodes and inguinal lymph nodes collected from five 45 days-age weaned piglets infection with PTTSuV2. With the development of infection, the sections of tonsil, spleen, hilar lymph nodes and mesenteric lymph nodes showed the normal architecture with no special lesions but only rarely red blood cells in the microvasculation and infiltration of inflammatory cells (lymphocytes and eosinophils) of tonsil and hilar lymph nodes marked by black arrowheads. In addition, inguinal lymph nodes' architecture was normal, but congestion, infiltration of inflammatory lymphocytes and necrosis or degeneration and focal gliosis of lymphocytes in the paracortical zone were observed.

## Discussion

In the experimental study, porcine TTSuV2 genome were identified in the serum of a conventional pig used as the source material of porcine TTSuV2 for inoculation into gnotobiotic porcine. TTSuV has been elucidated that it was readily transmitted to young gnotobiotic swine that had negative results for TTSuV genome [45], serial passage of liver tissue homogenate which was extracted twice with chloroform so as to remove infectivity of any extraneous enveloped viruses [45] obtained from the pig with positive result for porcine TTSuV2 and negative result for porcine TTSuV1 and PCV2 by using nPCR and conventional PCR. In pigs euthanized at 1, 3, 7, 10, 14, 17, 21 and 24 days post inoculation. The previous investigations have found a higher positive rate of porcine TTSuV in analyzed reproductive apparatus samples [37], indicating its importance in public hygienics and vertical transmission. Considering its transmission routine [30,46-48], specific pathogen-free pregnant primiparous sows were subjected to produce porcine TTSuVs negative specific pathogen-free piglets.

The present study evaluated the histopathological lesions of inoculation infection of the porcine Circoviridae family DNA virus torque tero sus virus (TTSuV) in main parenchmatous organs containing heart, lung, kidney, digestive system and immune system tonsil, spleen and lymph nodes on account of the viral tropism to lymphoid cells [45,49]. The results presented here specific histopathological lesions were conduced by porcine TTSuV2 infection alone in analyzed organs and tissues, there existed subtle pathologic changes in the tissues containing myocardial fibroklasts and endocardium, interstitial pneumonia, membranous glomerular nephropathy, and modest inflammatory cells infiltration in portal areas in the liver, foci of hemorrhage in some pancreas islet, a tiny amount red blood cells in venule of muscularis mucosae and outer longitudinal muscle, rarely red blood cells in the microvasculation and infiltration of inflammatory cells (lymphocytes and eosinophils) of tonsil and hilar lymph nodes, infiltration of inflammatory lymphocytes, necrosis or degeneration and focal gliosis of lymphocytes in the paracortical zone after inoculation with the porcine TTSuV2-containing tissue homogenate; these changes were not detected in uninoculated control pigs or pigs injected with tissue homogenate devoid of porcine TTSuV2 genome. Although TTSuV seems to be non-pathologic virus for the domestic pig [37], those results indicated it can induce a certain degree of lesions for some organs of pigs, and the present study was similar to the histopathological lesions via parenteral inoculation of g1-TTSuV-positive tissue homogenates into TTSUuV-negative gnotobiotes [45], so it is necessary to compare the different histopathological lesions caused by porcine TTSuVs between single-infection and co-infection. Through the investigation, we found that the main severely microscopic lesions happened at the respiratory system, urinary system and cardiovascular system, however, there existed only minimal injury in the digestive system and immune system, those results indicated no marked histopathological changes happened in those parenchymatous organs, especially the digestive system and immune system when the specific pathogen-free piglets were infected with porcine TTSuV2, hence we can think it was so poor pathological agent for domestic pigs at least.

To date, it has been found that it is common for an individual to coinfect with different genogroups of TTSuV [3] and the role of porcine TTSuV in co-infection with other pathogens has been investigated and demonstrated porcine TTSuV2 is frequently related to PCV2 associated diseases (PCVAD) while compared to porcine TTSuV1 in Spain [18]. In the swine industry, TTSuV is thought to be one of the agents that aggravate clinical manifestation of porcine circovirus-associated disease (PCVAD), a newly emerging, economically devastating disease. However, a researcher verified there were no statically considerable differences in TTSuV viral load between the two genotypes in serum obtained from porcine circovirus-2-negative pigs and PCVAD-affected pigs with real-time quantitative polymerase chain reaction assay, which indicated that TTSuV might not be an etiology of aggravation in PCVAD, and TTSuV genogroup 2 could readily give rise to viremia even in the PCV-2-negative pigs [16]. Other reports indicated thatTTSuV1 viral infection facilitated PCVAD [36]. Interestingly, positive stillborn piglets were always positive with the same genotype as their mothers, but sequencing analysis showed nucleotide diversity of TTSuV genomes from the sows and in stillborns [30]. It was hypothesized that stillborns may be infected with several strains of TTSuV, even from the semen, by the transpla cental route [30]. Higher rate of PCV2 DNA in semen (47% of tested samples) and followed by TTSuV2 (11.7%) has been investigated in spite of presenting with a low viral load and all tested semen samples were negative for TTSuV1 [37]. Other investigations published a higher prevalence of TTSuV1 and TTSuV2 in pig semen (55% and 32%, respectively), with no interference on semen quality [47].

Viral persistence and sporadic infection may be a risk factor for dissemination of PCV2 or TTSuV to negative sows, potentially resulting in reproductive failures. This irregular pattern of infection also paid attention to the fact that boars which tested negative on the first collection may be positive in subsequent ejaculates. A periodic monitoring for PCV2 or TTSuV2 must be established in boar community. PCV2 and TTSuV2 presence in semen samples of younger animals may indicate a recent infection or may even be related to management or measures of stress. Morphology and sperm motility analysis did not indicate significant diversities between PCV2 positive and negative boars. All semen samples tested presented motility superior to 80%, which could allow their use for processing, dilution and artificial insemination. No manifestation of reproductive failure was associated with this co-infection. These findings raised the problem of the importance of these viral infections in the pathology of reproductive failures [37]. Although TTSuV2 was detected in almost 50% of the sows studied, the association with PCV2 co-infection and reproductive failure was statistically insignificant difference [37].

Moreover, in both species, genotype 2 is more prevalent than genotype 1. In domestic pig, TTSuV2 infections have been shown to be more common in pigs affected by postweaning multisystemic wasting syndrome (PMWS), a porcine circovirus type 2 (PCV2) disease, than non-PMWS affected pigs [18,49]. Due to its ubiquitous nature in both domestic pig and wild boar, it is likely that TTSuV has adapted to both species and is circulating within these species with similar prevalence [3]. On account of lack in a culture system or an animal model to support the viral multiplication, the infection and replication mechanisms and the pathogenicity of TTSuV are still unknown. Some reports have indicated that several different genotypes of TTSuV are considered responsible for human diseases [50]. However, TTSuV has been indicated to be a commensal in normal conditions which should benefit for the host, this is an intriguing aspect hitherto unexplored with TTSuV [51]. In 2006, a surprising finding was found some marked differences in prevalence of TTSuV genotype 2 were induced by age, that is to say that younger animals were more often infected than adults and sub-adults, it would be interesting to compare such dynamics in wild boar with that in domestic pig since the same agents apparently infect both species, prevalence of both TTSuV genotypes was higher in females than males, although only important for genotype 2. In addition, there lack in comparison patterns with domestic pigs or other species that suffer from TTSuV infection [3].

Up to now, to our knowledge, the only available method for detection of TTSuV is focused on nucleic acid detection, such as nested polymerase chain reaction (nPCR) [18], quantitative PCR (qPCR) [16], and enzyme-linked immunosorbent assay (ELISA) based on ORF1 of porcine TTSuV2 has presently been investigated but still not has been applied clinically [44]. To date, no tissue culture system and little information for detection method of pathology has been available for the propagation of the virus. Further study of TTSuV pathology is required to answer those lesions of single infection of porcine TTSuV or co-infection with other agents, and diverse technologies are required for defining the role of TTSuV in clinics and public health. The pathogenesis of porcine TTSuV infection and its link with specific diseases are as yet undetermined. This result maybe be considered as important descriptive for the histological lesions caused by infection with porcine TTSuV type 2. In this study, it, to some extent, was lack of scientific by using liver homogenate containing porcine TTSuV2 genome to inoculate the specific pathogen-free piglets, because it was difficult to rule out others non-enveloped viral genomes contamination, despite non-infecting agents were isolated via marc145 and vero cells to purify the homogenate in our previous study, so we propose the primary task of researchers is to seek a new cell culture system to separate and isolate porcine torque teno sus virus.

## Conclusions

Analysis of these presentations revealed that porcine TTSuV2 was readily transmitted to TTSuV-negative swine and that infection was associated with characteristic pathologic changes in specific pathogen-free piglets inoculated with porcine TTSuV2. Those results indicated no markedly histopathological changes happened in those parenchymatous organs, especially the digestive system and immune system when the specific pathogen-free pigs were infected with porcine TTSuV2, hence, to some extent, it was not remarkable pathological agent for domestic pigs at least. So, porcine TTSuV2 could be an unrecognized pathogenic viral infectious etiology of swine. This study indicated a directly related description of lesions responsible for TTSuV2 infection in swine.

## Methods

### Experimental animals

Date-mated specific pathogen-free pregnant primiparous sows fetched in Canada were purchased from a new constructed pigfarm located in Chongqing and transported to National Animal Experimental Teaching Demonstration Center of Sichuan Agricultural University for caesarian section of specific pathogen-free piglets litters using methods described elsewhere [52]. Twenty-four 28-day-old specific pathogen-free piglets which were screened for the absence of PCVs, genogroup 1 and genogroup 2-TTSuV DNA through nested PCR used for histopathological examination for porcine infection with TTSuV genogroup 2.

### Source of virus and detection

Porcine torque teno sus virus 2 was derived from 20% (w/v) liver tissue homogenate which was extracted twice with chloroform so as to remove infectivity of any extraneous enveloped viruses [45] and was designated as infection with porcine TTSuV2 by nPCR not with PCV2 by use of PCR assay, and collected from a 6-week-old clinically asymptomatic pig and tested negative for classic swine fever virus (CSFV), porcine peproductive and respiratory syndrome virus (PRRSV), porcine pseudorabies virus (PRV), Japanese encephalitis virus (JEV), porcine parvovirus (PPV), transmissible gastroenteritis virus (TGEV), porcine circovirus type 2 (PCV2), swine influenza virus (SIV), porcine cytomegalo virus (PCMV) by IFA, RT-PCR, multiple-PCR, nPCR or conventional PCR based on those viral genomic un-translation regions or specific genes, and the aqueous phase was frozen at -70°C by Animal Biotechnological Center of Sichuan Agricultural University, and the viral DNA loads (6.91 × 10^7^genomic copies/ml) is determined by SYBR green-based real-time quantitative PCR.

### Experimental design

Twenty-four 28-day-old specific pathogen-free piglets were randomly divided two groups, housed separately in the experimental animal houses at National Animal Experimental Teaching Demonstration Center of Sichuan Agricultural University, twenty-one piglets used as experimental group which were inoculated by intramuscular route with 1 ml of 6.91 × 10^7^genomic copies viral loads of porcine TTSuV2 liver tissue homogenate and humanely killed at 3,7,10,14,17,21 and 24 days post inoculation (dpi). The three remaining pigs, which were used as control pigs, were injected intramuscularly with 1 ml of sterile Dulbecco's modified Eagle's medium (DMEM, Gibco, USA), supplemented with 10% (v/v) fetal calf serum (FCS, Gibco, USA), 100 IU/ml of streptomycin and penicillin, respectively, and humanely killed the end of the study.

### Histopathological examination

All pigs were subjected to gross necropsy examination and main tissues and organs samples for microscopical examination were collected from each pig at the same time of necropsy. These samples contained portions of heart, liver, spleen, lung, kidney, hilar lymph nodes, mesenteric lymph nodes, inguinal lymph nodes, pancreas, tonsil and duodenum for histopathological examination. All tissues were fixed in 10% neutral buffered formalin for 24-48 hours and routinely processed and embedded in paraffin wax. Sections were cut into slices with 4 ~ 5 micrometer thick and stained with hematoxylin and eosin (HE). Those slices were detected under a light microscope for conventional morphological evaluation.

## Competing interests

The authors declare that they have no competing interests.

## Authors' contributions

MM, LZ carried out most of the experiments and drafted the manuscript. ZWX and WZG critically revised the manuscript and the experimental design. YW, XP, LZ, YFW and SL helped with the study. All of the authors read and approved the final version of the manuscript.
